# Parlamentarische Technikfolgenabschätzung und Risikokommunikation

**DOI:** 10.1007/s00103-022-03523-0

**Published:** 2022-04-06

**Authors:** Armin Grunwald

**Affiliations:** Institut für Technikfolgenabschätzung und Systemanalyse (ITAS), Karlstr. 11, 76133 Karlsruhe, Deutschland

**Keywords:** Parlamente, Inklusion, Partizipation, Deliberative Demokratie, Vorsorge, Parliaments, Inclusion, Participation, Deliberative democracy, Precaution

## Abstract

Mit der parlamentarischen Technikfolgenabschätzung (TA) hat sich in vielen Ländern weltweit ein eigener Typus wissenschaftlicher Politikberatung entwickelt. In Deutschland besteht das Büro für Technikfolgenabschätzung beim Deutschen Bundestag (TAB) seit über 30 Jahren. Zur Entstehungsgeschichte und Gegenwart parlamentarischer TA gehört die Erfahrung nichtintendierter Folgen des wissenschaftlich-technischen Fortschritts, speziell das Auftreten von technikbedingten Risiken, untrennbar hinzu. Von Beginn an ist die Perspektive der TA auf Risiken durch differenzierte Risikowahrnehmung gekennzeichnet, vor allem in Bezug auf unterschiedliche Bewertungen durch die Entscheider, Nutznießer, Stakeholder und die möglicherweise von Gefährdungen und Belastungen Betroffenen. Daher spielt die soziale Dimension der Verteilung von Chancen und Risiken zwischen gesellschaftlichen Gruppen, aber auch zwischen heute lebenden Menschen und zukünftigen Generationen, eine wesentliche Rolle. Die TA gibt, basierend auf Prinzipien deliberativer Demokratie, der Inklusion von Betroffenen mit ihren Einschätzungen, Werten, Wissen und Perspektiven wesentlichen Raum in Risikodebatten. In diesem Beitrag werden zunächst die Motivation und Aufgaben der TA generell und von parlamentarischer TA im Besonderen vorgestellt, um sodann speziell auf den Umgang mit nichtintendierten Folgen und Risiken einzugehen. Auf Basis von Erfahrungen aus früheren Risikodebatten, etwa zur Nanotechnologie, zur Endlagerung hochradioaktiven Abfalls und zur grünen Gentechnik, werden Schlussfolgerungen für moderne Risikokommunikation auf politischer Ebene gezogen.

## Abstract (en)

Parliamentary technology assessment (TA) has developed in many countries worldwide as a specific approach to scientific policy advice. In Germany, for example, the Office of Technology Assessment at the German Bundestag (TAB) has been serving the national parliament for more than 30 years. The occurrence of unintended side effects of the scientific and technological advance, in particular of technology-induced risks, has been a major motivation of TA from its beginning until present. Its perspective on risks can be characterized by a pluralist approach observing differentiated perception and evaluation of technology-induced risks among different social groups. In particular, risk perception frequently differs between decision-makers, benefiters, stakeholders, and groups affected by possible hazards and stresses. Therefore, the social dimension of the distribution of chances and risks and their differentiated perception among various societal groups as well as between humans living today and future generations, play a major role in risk assessment by TA. Based on general ideas of deliberative democracy, TA has therefore developed approaches to involve people affected with their partially diverging risk perceptions, values, knowledge, and perspectives. In this article, the motivation and mission of TA in general, and of parliamentary TA in particular, will be introduced. The article focuses on the TA principles of inclusion and participation involving societal groups, stakeholders, and citizens in risk assessment. Lessons learned from previous risk debates, such as on nanotechnology, nuclear waste disposal, and green biotechnology, are exploited for deriving conclusions for modern risk communication at the political level.

## Zielsetzung und Überblick

Die Technikfolgenabschätzung („technology assessment“; TA) ist vor etwa 50 Jahren als wissenschaftliche Politikberatung zu Fragen des technischen Fortschritts entstanden [1]. Von Anfang an spielten in der TA technikbedingte Risiken als nichtintendierte, häufig auch nicht vorhergesehene und zumeist unerwünschte Technikfolgen eine zentrale Rolle [2], um Chancen des technischen Fortschritts bestmöglich nutzen, Risiken minimieren und mit den verbleibenden verantwortlich umgehen zu können. Vielfach jedoch treten Bewertungsunterschiede zwischen Entscheidern, Nutznießern, Stakeholdern, Bürgern und möglicherweise von Schäden Betroffenen, aber auch antizipierten zukünftigen Generationen im Fall der nachhaltigen Entwicklung auf. Während in vielen Chance-Risiko-Bilanzierungen volkswirtschaftlich oder statistisch auf hoch aggregierter Ebene argumentiert wird, räumt die TA der *Verteilung* von Chancen und Risiken zwischen gesellschaftlichen Gruppen wesentlichen Raum ein.

Die charakteristische Perspektive der TA für Risikokommunikation und Risikobewertung ist durch Einbeziehung differenzierter und häufig auch deutlich divergierender Risikowahrnehmungen gekennzeichnet. Dabei nutzt sie Prinzipien deliberativer Demokratie [3], insbesondere der *Inklusion* von Betroffenen mit ihren Einschätzungen, Werten, Wissen und Perspektiven in partizipativen Verfahren [4], um in Risikodebatten erstens ein umfassendes Bild zu gewinnen, um zweitens mögliche Deeskalations- und Verständigungsbereiche zu identifizieren, um drittens möglichst robuste Strategien zur Nutzung von Chancen und zum verantwortlichen Umgang mit Risiken zu entwickeln (siehe Abschnitt „Technikfolgenabschätzung: Technikrisiken und Partizipation“) und schließlich um zu einem sachorientierten Dialog in der Gesellschaft beizutragen.

Die parlamentarische TA [5] ist in vielen Ländern als ein eigener Typus wissenschaftlicher Politikberatung etabliert [6]. In Deutschland beispielsweise besteht das Büro für Technikfolgenabschätzung beim Deutschen Bundestag (TAB) seit über 30 Jahren [7, 8]. Die unmittelbare Nähe zum politischen Geschehen sowie die Verpflichtung zu wissenschaftlicher Unabhängigkeit und Zurückhaltung in Bewertungsfragen haben für Risikoanalysen und Risikokommunikation ein spezielles Umfeld geschaffen, durch das die entsprechenden Institutionen in ihrer Risikokommunikation geprägt sind (siehe Abschnitt „Parlamentarische Technikfolgenabschätzung und Risikokommunikation“).

In diesem Beitrag wird zunächst die Ausgangskonstellation der TA im Allgemeinen vorgestellt, wobei der Schwerpunkt auf dem partizipativen und inklusiven Umgang mit nichtintendierten Folgen und Risiken liegt (siehe Abschnitt „Technikfolgenabschätzung: Technikrisiken und Partizipation“). Der Fokus auf der parlamentarischen TA verlangt, die spezifische Arbeitsweise und Beratungssituation an Parlamenten mit einem engen Politikbezug kurz zu erläutern (siehe Abschnitt „Parlamentarische Technikfolgenabschätzung und Risikokommunikation“). Das hier geltende starke Unabhängigkeits- und Neutralitätsgebot kanalisiert die Möglichkeiten ihrer institutionellen Risikokommunikation und verlagert sie zu einem Teil auf die Beratungsleistung an die jeweiligen Parlamente. Erfahrungen aus früheren Risikodebatten mit Beteiligung der parlamentarischen TA, etwa zur Nanotechnologie, zur Endlagerung hochradioaktiven Abfalls und zur grünen Gentechnik (siehe Abschnitt „Lehren aus früheren Risikodebatten“), werden schließlich für Schlussfolgerungen für moderne Risikokommunikation auf politischer Ebene herangezogen.

## Technikfolgenabschätzung: Technikrisiken und Partizipation

Wissenschaft und Technik haben mit großen Fortschritten in Medizin und Gesundheit, Mobilität und Energieversorgung und vielen anderen Bereichen Lebensqualität und Wohlstand und damit die moderne Gesellschaft ermöglicht. Jedoch sind spätestens seit den 1960er-Jahren erhebliche nichtintendierte Technikfolgen in teils dramatischen Ausprägungen unübersehbar. Durch Großunfälle in technischen Anlagen (z. B. in Seveso, Bhopal, Tschernobyl, Fukushima), die globale Umweltkrise (z. B. Biodiversitätsverlust, Mikroplastik, Klimawandel), Arbeitsmarktprobleme als Folge der Automatisierung, Risiken für die Demokratie durch bestimmte Effekte der Internetkommunikation, ethische Herausforderungen von Gen- und Biotechnologien sowie Dual-Use-Möglichkeiten (Produkte, Software und Technologie, die zivil, aber auch militärisch genutzt werden können) und Missbrauchspotenziale von Technik auf unterschiedlichen Ebenen haben sich allzu fortschrittsoptimistische Zukunftserwartungen als naiv erwiesen ([1], vgl. bereits [9]). Angesichts dieser *Ambivalenz* der Technik [10] sind Technikentwicklung und -einführung nach dem Prinzip von Versuch und Irrtum weder politisch oder ökonomisch rational noch ethisch verantwortbar, sondern ist die *vorausschauende* Analyse und Bewertung von Technikfolgen unerlässlich geworden.

Diese Feststellung war bereits bekannt als die Technikfolgenabschätzung (TA) vor etwa 50 Jahren im US-amerikanischen Kongress entstanden ist [11] und sich von dort aus zu einer internationalen Forschungs- und Beratungsdisziplin entwickelt hat [1]. Sie befasst sich antizipativ mit dem gesamten Spektrum von Technikfolgen einschließlich nichtintendierter Folgen und möglicher Gefährdungen. Das dadurch erzeugte Wissen dient dazu, Gesellschaft und Entscheidungsträger zu beraten, um umfassend informierte und reflektierte Entscheidungsfindung zu unterstützen, so etwa in der Energiewende, der Digitalisierung oder in Bezug auf neue Mobilitätssysteme.

In dieser Ausrichtung ist die TA eine der Antworten der Wissenschaft auf die Notwendigkeit einer reflexiven Modernisierung [12] angesichts der Diagnose der Risikogesellschaft [13] und des unvermeidbaren Auftretens systemischer und komplexer Nebenfolgen [14]. In der Risikogesellschaft sind immer mehr Entwicklungen direkt oder indirekt mit menschlichen Handlungen und Ursachen verwoben und damit mittelbar oder unmittelbar Folgen menschlicher Intervention. Beispielsweise werden die Folgen eines Vulkanausbruchs stark vom menschlichen Siedlungsverhalten in der Umgebung mitbestimmt. Auch die Fukushima-Katastrophe ist mit der Jahrzehnte früher getroffenen Entscheidung verbunden, die Kraftwerksblöcke gerade dort zu installieren und sie für bestimmte Stärken von Erdbeben und bestimmte Höhen von Tsunamis auszulegen. Ursachen der Havarie waren dementsprechend nicht einfach Erdbeben und Tsunami, sondern menschliche Entscheidungen über Standort und Design der Anlagen. Diese Überlegung verdeutlicht die mit technikbezogenen Entscheidungen verbundene Verantwortung [9], so etwa zum autonomen Fahren [15] oder zu der Freisetzung von Nanopartikeln oder Chemikalien.

Risiken enthalten 3 konstitutive Bedeutungselemente [15]: (1) *Unsicherheit* angesichts der bloßen Möglichkeit des Eintretens von Schäden, (2) *Unerwünschtheit* angesichts ihres Schadenscharakters und der damit verbundenen Negativbewertung sowie (3) das *soziale Moment* der Verteilung von Chancen und Risiken auf verschiedene Personen oder Gruppen.

Die Unsicherheit führt zu der Frage, was über mögliche nichtintendierte Folgen (Eintrittswahrscheinlichkeit, Schadensart und -größe) gewusst wird und wie belastbar dieses Wissen ist. Die Unerwünschtheit verweist auf den Wunsch der weitestgehenden Vermeidung von Schaden in der Nutzung der Chancen. Die Bewertung von möglichen Folgen des Handelns als Risiko oder als Chance kann durchaus umstritten sein, je nachdem, ob für bestimmte Gruppen der Chancen- oder der Risikoaspekt überwiegt. Dieser Perspektivbezug führt auf das soziale Moment: Risiken sind immer Risiken *für jemanden* bzw. für bestimmte Akteure. Die von bestimmten Handelnden intendierten und damit erwünschten Folgen müssen keineswegs auch bei anderen erwünscht sein, sondern können dort Belastungen, Belästigungen oder gar Gefährdungen bedeuten. Dies ist häufig eine Ursache für Konflikte, etwa in Standortfragen technischer Anlagen oder in der Chemikalienbewertung. Insbesondere die in der Wahrnehmung und Bewertung dieser Risiken häufig auftretende Differenz zwischen der Entscheider- und der Betroffenenperspektive ist für die TA von zentraler Bedeutung [16].

Dieser sozialen Dimension des Risikos kommt in gesellschaftlichen Technikdebatten und -kontroversen eine zentrale Bedeutung zu. Die volkswirtschaftlich-statistische Argumentation, etwa zur Zahl neu geschaffener Arbeitsplätze, trifft häufig nicht die Risikosensibilität auf der Ebene von Gruppen, Regionen, Kommunen, Stakeholdern oder Bürgern. Dass eine neue Start- und Landebahn an einem Flughafen neue Wertschöpfungspotenziale für die Region erschließt, ist aus Perspektive vieler, jedoch sicher nicht aller Anwohner wenig oder gar nicht relevant. Dass mit der Industrie 4.0 neue Arbeitsplätze im IT-Bereich entstehen, bringt denjenigen Berufsgruppen nichts, die dabei ihre Arbeitsplätze durch Automatisierung verlieren und für die neuen Arbeitsplätze nicht qualifiziert werden können. Bei Abwägungen von Chancen und Risiken muss daher immer auch gefragt werden, wer von den Risiken wie betroffen ist, wer von den Chancen wie profitiert und ob mehr Verteilungsgerechtigkeit erreicht werden kann. Gerade diese Thematik ist für die Risikokommunikation entscheidend.

In der Governance von Risiken hat die TA frühzeitig zwischen Entscheider- und Betroffenenperspektive zu unterscheiden gelernt, die häufig auch die Grenze zwischen Chancenerwartungen und Risikobefürchtungen markiert [16]. In einer etwas härteren Wortwahl bedeutet dies letztlich, dass Abwägungen zwischen Chancen und Risiken auf der sozialen Seite letztlich oft Gewinner-Verlierer-Konstellationen mit sich bringen: Einige profitieren, anderen wird etwas zugemutet [17]. Hier stellen sich deswegen die Fragen, nach welchen Kriterien die Abwägungen vorgenommen werden, in welchen Verfahren dies geschieht, welche Akteure darüber letztlich entscheiden und wie diese dafür legitimiert sind. Gruppen, denen aus übergeordneten Gründen Risiken zugemutet werden, wollen schließlich verstehen, warum gerade sie diese „Verlierer“ sein und das entsprechende Risiko oder zumindest die Belastungen akzeptieren sollen, etwa bei Standortfragen technischer Anlagen oder bei der Trassenführung von Hochspannungsleitungen [17].

Die traditionell häufige Konfrontation zwischen Entscheidern, die nicht den Risiken ausgesetzt sind, sondern die Chancen sehen, und den Betroffenen, für die die Chancen abstrakt, die Risiken aber konkret sind, ist vielfach unter den Stichworten Akzeptanzverweigerung oder Technikablehnung behandelt worden, häufig verbunden mit der Suche nach Verfahren der „Akzeptanzbeschaffung“ [17]. Dies ist aus Sicht der TA eine falsche Herangehensweise, die den unterschiedlichen und auf ihre Weise gleichermaßen legitimen Perspektiven nicht gerecht wird [18]. Stattdessen hat sich die TA seit den 1980er-Jahren in Richtung Partizipation und Mitwirkung im gesellschaftlichen Dialog geöffnet [4], um die Betroffenen nicht als passive Gruppe zu betrachten, die für etwas überzeugt oder zu etwas überredet werden müsse, sondern um sie als aktive Teilnehmer in die Technik- und Risikobewertung einzubeziehen.

Der amerikanische Philosoph John Dewey [19] gilt als ein Vordenker dieser Ausrichtung der TA [1]. Nach seiner politischen Philosophie besteht die wesentliche Aufgabe der Politik darin, den Umgang mit Folgen und Risiken des in möglichst großer Freiheit erfolgenden Handelns der individuellen Menschen zu regulieren.

Diese Bestimmung ist unmittelbar verknüpft mit der Aufgabe der TA, gerade die nichtintendierten Folgen und insbesondere Risiken des wissenschaftlich-technischen Handelns zu erforschen und verantwortliche Handlungsstrategien zu entwickeln. Entsprechend arbeitet die TA seit Jahrzehnten zunehmend transdisziplinär mit unterschiedlichsten Gruppen und Organisationen der Zivilgesellschaft zusammen [20], um die sachliche und politische Legitimation von Risikobewertungen zu verbessern, aber auch um das spezifische Wissen zivilgesellschaftlicher Gruppen einzubringen [4, 20]. Letztlich besteht die Erwartung, dadurch robustere Bewertungen und Handlungsstrategien zu ermöglichen. Hier zeigt sich auch die Ausrichtung der TA auf einen demokratischen Gestaltungsanspruch im Umgang mit dem wissenschaftlich-technischen Fortschritt und seinen Folgen und möglichen Gefährdungen bzw. Belastungen, denn die Abwägung von und Entscheidung über Chancen und Risiken trägt genuin politischen Charakter. Einem technokratischen „science knows best“ (kritisch dazu [21]) in Fragen der Risikobewertung, etwa durch modellbasierte Optimierung, stellt die TA ein Denken in alternativen Optionen als Modus wissensbasierter, aber demokratischer Deliberation entgegen [22].

## Parlamentarische Technikfolgenabschätzung und Risikokommunikation

Die TA als wissenschaftliche Politikberatung an Parlamenten ist in den 1960er-Jahren am US-amerikanischen Kongress entstanden [11]. Der Zeitpunkt korreliert mit der zunehmenden Bedeutung des Staates in der Technologie- und Forschungspolitik, der stark wachsenden Bedeutung von Wissenschaft und Technik im Wirtschaftswunder der Nachkriegszeit genauso wie im Kalten Krieg, aber auch mit der verstärkten Bewusstwerdung nichtintendierter Technikfolgen, vor allem im Umweltbereich.

In dieser Situation sah der US-Kongress sich zusehends nicht mehr in der Lage, auf Basis eigener Expertise seinen Aufgaben nachzukommen. Die TA wurde etabliert, um den Kongress als entscheidungsfähige und kompetente Volksvertretung auch in Fragen von Wissenschaft und Technik zu stärken. Die zentralen Aufgaben des 1972 als wissenschaftliche Beratungskompetenz gegründeten *Office of Technology Assessment* (OTA; [11]) waren [23]: Frühwarnung vor Risiken genauso wie Früherkennung von Chancen, Ausarbeitung von alternativen Lösungswegen einschließlich der Abschätzung der jeweiligen Konsequenzen und Implikationen und Bündelung der Ergebnisse für politische Entscheidungsprozesse. Dies geschah durch Einbeziehung von externem Sachverstand und sollte das Vertrauen der Öffentlichkeit in die Legitimität politischer Entscheidungen stärken.

Dieses Programm wurde – wenngleich das OTA bei Weitem nicht alle diese Aufgaben vollumfänglich realisieren konnte – zum Vorbild parlamentarischer TA bis heute [1]. So wurden ab der zweiten Hälfte der 1980er-Jahre in mehreren europäischen Ländern Einrichtungen parlamentarischer TA gegründet. Die Zahl entsprechender Einrichtungen weltweit wächst langsam, aber stetig, ihr Zuschnitt ist entsprechend den nationalen Besonderheiten sehr unterschiedlich, z. B. in Bezug auf Arbeitsweise und Rolle der Parlamente und die jeweiligen Zuständigkeiten im Bereich der *Technology Governance* [24]. Heute sind 22 Institutionen parlamentarischer TA im *European Parliamentary Technology Assessment Network* (EPTA) zusammengeschlossen. Zunehmend schließen sich auch Staaten darüber hinaus wie Südkorea, Japan, die USA und Chile an.

In Deutschland wurde das TAB 1990 gegründet [7]. Alle 5 Jahre wird vom Bundestag über den Weiter- oder Neubetrieb durch eine externe Forschungsinstitution entschieden. Seit 1990 wird das TAB vom Karlsruher Institut für Technikfolgenabschätzung und Systemanalyse (ITAS) betrieben, immer wieder mit anderen Partnern und anderen Schwerpunkten.

In dieser Zeit wurden über 200 Studien für den Bundestag erstellt, darunter eine ganze Reihe zu Gesundheitsfragen. Über die Themen entscheidet der Bundestag als Auftraggeber und Geldgeber nach Beratungen mit dem TAB über den wissenschaftlichen Neuigkeitswert, die gesellschaftliche Relevanz und den möglichen politischen Nutzen einer TA-Studie [25]. Für das TAB betreffende Beschlüsse gilt das* Konsensprinzip*, was in den Arbeitsabläufen des Bundestages sehr ungewöhnlich ist. Die Bearbeitung der ausgewählten Themen erfolgt durch das TAB in strikter wissenschaftlicher Unabhängigkeit und politischer Neutralität im Sinne von Unparteilichkeit und Unvoreingenommenheit mittels Einbeziehung fachlicher Expertise, etwa aus Universitäten und Forschungseinrichtungen.

Die inhaltliche und institutionelle Unabhängigkeit ist ein wesentlicher Grundpfeiler parlamentarischer TA. Das semantische Feld des Begriffs wissenschaftlicher Unabhängigkeit umfasst verwandte oder teils synonym verwendete Begriffe wie Neutralität, Unvoreingenommenheit, Werturteilsfreiheit, Sachlichkeit, Objektivität, Unparteilichkeit oder Ausgewogenheit [26]. Die Finanzierung durch öffentliche Gelder und die Beauftragung durch das gesamte Parlament statt durch Parteien haben dies zur notwendigen Folge. Auch aus Klugheitsgründen muss die parlamentarische Basis möglichst breit sein und im Idealfall alle Fraktionen umfassen, um Überlebenschancen über Mehrheitswechsel hinweg zu ermöglichen.

Dies war bereits beim US-amerikanischen OTA ein Thema. Dort befürchteten die Republikaner stets die Dominanz der Demokraten. Als die Republikaner in beiden Kammern des Kongresses die Mehrheit hatten, wurde das OTA abgeschafft [11]. Die Verpflichtung auf wissenschaftliche Unabhängigkeit ist daher zentral für parlamentarische TA-Einrichtungen [26]. Im Falle des TAB ist sie auch vertraglich festgeschrieben. Ihre Realisierung ist unabdingbar, um in politischen und öffentlichen Diskussionen, insbesondere zu Risikokontroversen, als vertrauenswürdig wahrgenommen zu werden.

Viele Studien des TAB stehen in direktem Zusammenhang mit Fragen von Risiko und Sicherheit. In der Anfangszeit des TAB waren dies vor allem Studien im Kontext der Gentechnik, in denen es gelang, das TAB trotz der damals hochkochenden Risikodebatte als auf allen Seiten anerkannten und unabhängigen Akteur zu positionieren [27]. Immer wieder reichten die Aufträge des Bundestages bis hin zu Grundfragen des operativen Risikomanagements, so etwa zum Nachzulassungsmonitoring transgener Pflanzen [28] und zur Risikoregulierung bei unsicherem Wissen [29].

Weitere Felder risikobezogener TAB-Studien sind der Mobilfunk, die bereits erwähnte Nanotechnologie, Technik- und Transformationsthemen der Energiewende, die Reproduktionsmedizin, die synthetische Biologie, das genetische Editieren und andere Felder moderner Biotechnologie sowie in den letzten Jahren zahlreiche Themen aus dem Feld der Digitalisierung. Themen aus dem Gesundheitsbereich haben das TAB ständig begleitet, hier seien beispielhaft die Studien zur individualisierten Medizin [30], zur Fortpflanzungsmedizin [31] und zu Gesundheits-Apps [32], also zu einem der zahlreicher werdenden Berührungspunkte zwischen Medizin und Digitalisierung genannt. Hier wurde jeweils die Risikoanalyse und -bewertung in der gesellschaftlichen wie auch in der sozialen Dimension (s. oben) berücksichtigt.

Angesichts der Konflikthaftigkeit vieler Risikothemen, wie etwa Kernenergie, 5G-Mobilfunk oder gentechnisch veränderter Nahrungsmittel, stellen wissenschaftliche Unabhängigkeit und Neutralität parlamentarischer TA eine Herausforderung an die Risikokommunikation dar, da sie eine substanzielle Festlegung in Bewertungsfragen erschweren. Stattdessen operieren parlamentarische Einrichtungen häufig in einem Wenn-dann-Modus: Wenn bestimmte Bewertungskriterien herangezogen werden und wenn spezifische Annahmen gemacht werden und wenn …, dann ist das Risiko wie folgt einzuschätzen und zu bewerten. Dieser Modus entspricht letztlich wissenschaftlich-argumentativem Denken und der dementsprechenden Arbeitsweise. Er erlaubt ebenfalls die partizipative Einbeziehung unterschiedlicher Perspektiven (siehe Abschnitt „Technikfolgenabschätzung: Technikrisiken und Partizipation“) in diese Exploration von Risiken, Wahrnehmungen und Einschätzungen.

Auf diese Weise wird eine Art Kartierung der Risikokonstellation vorgenommen, ohne eine substanzielle Festlegung auf eine bestimmte Position – diese liegt im Mandat der politischen Parteien bzw. des Deutschen Bundestages. Dieses Verhältnis von wissenschaftlicher TA und politischer Bewertung wurde treffend in folgendem Bild dargestellt:

… researchers, along with stakeholders, act as the „cartographers“ of different, viable policy pathways and their practical consequences by acting as the „mapmakers“ of the political solution space. They provide a guidebook with alternative options for policymakers (i.e., the „navigators“ and the public). Such maps cannot replace travelling i.e., decision-making nor can they resolve all environmental policy conflicts, yet they can provide an important orientation in otherwise uncharted territory [33, S. 63].

Dieses Modell ist spezifisch für wissenschaftliche Politikberatung an Parlamenten und unterscheidet sich kategorial etwa vom Risikohandeln hoheitlicher Organisationen und Behörden. Deren Aufgabe ist es, substanzielle Festlegungen zu Risikofragen, etwa zu Grenzwerten und Zulassungen, vorzunehmen und damit praktisch wirksam zu werden. Um im Bild zu bleiben: In der Kartierung von Risikokonstellationen, die alternative Möglichkeiten der Festlegung enthält, müssen Entscheidungen für bestimmte Optionen getroffen und umgesetzt werden.

Hieraus ist zunächst zu erkennen, dass parlamentarische TA und operative Institutionen an unterschiedlichen Stellen in Risikodebatten arbeiten. Die TA operiert zu Fragen, bei denen die Risikokonstellation, z. B. im Gesundheitsbereich, meist noch unklar ist, wo über Bewertungskriterien, Vergleichsmaßstäbe, Verfahren der Bewertung und des Managements von Risiken noch hohe Unsicherheit besteht und ein politisch legitimierter Weg dazu erst noch gefunden werden muss (z. B. [30, 31]).

Das Risikohandeln von Behörden, etwa in Lebensmittelkontrolle, Arzneimittelzulassung oder Seuchenbekämpfung, ist dagegen durch die Umsetzung eines politisch legitimierten Auftrags gekennzeichnet, der sodann in wissenschaftlicher Unabhängigkeit bearbeitet wird. Während also die parlamentarische TA in differenzierter Kommunikation die Optionen im Umgang mit noch meist schlecht erkannten Risiken eruiert – dies häufig mit Stakeholdern und Zivilgesellschaft – und sich daher einer substanziellen Festlegung, etwa ob und wie ein Risiko verantwortlich übernommen werden kann, enthalten kann und muss, ist die behördliche Arbeit zu Risiken gerade durch Letzteres gekennzeichnet. Dies führt bis hin zum Treffen von Entscheidungen z. B. über Genehmigungen technischer Anlagen oder Chemikalienbewertungen im Rahmen des REACH-Systems.

Ein gutes Beispiel hierfür lässt sich in der politischen und wissenschaftlichen Verarbeitung von Risiken der Nanotechnologie in der frühen sogenannten Nano-Debatte vor etwa 20 Jahren erkennen. Diese Debatte war zunächst durch visionäre, jedoch hoch spekulative Erwartungen wie Befürchtungen geprägt, die zwischen Paradieshoffnungen und apokalyptischen Sorgen vor einem Ende der Menschheit oszillierten. Die weltweit erste TA-Studie [34], dem Deutschen Bundestag 2003 vorgelegt, machte die damals erst beginnenden toxikologischen Fragen nach dem Verbleib und möglichen Folgen von Nanomaterialen und Nanopartikeln im menschlichen Körper, später dann auch in der natürlichen Umwelt, zum Thema der öffentlichen wie politischen Wahrnehmung [35]. Sie explorierte erste Schritte zu einer Vorsorgeorientierung in Entgegensetzung zu den gleichzeitig publizierten ersten Forderungen nach einem Moratorium für Nanopartikel, bis deren Ungefährlichkeit erwiesen sei [36]. Aus diesen noch im Bereich hohen Nichtwissens über Nanopartikel und in Unsicherheit über adäquate Bewertungsschemata und Vergleichsmaßstäbe (immer wieder wurde damals die Parallele zu Asbestfasern gezogen) operierenden Ansätzen, mögliche Gefährdungen bzw. Belastungen einer operativen Regulierung zugänglich zu machen, wurde vor allem die Dringlichkeit von medizinischer und toxikologischer Forschung abgeleitet. Diese wurde vom Bundestag aufgegriffen und das Bundesministerium für Bildung und Forschung (BMBF) zur Förderung der neu entstehenden Nanotoxikologie aufgefordert.

In der Zwischenzeit sind Nanopartikel längst kein Thema parlamentarischer TA mehr, sondern etablierter Bestandteil behördlicher Chemikalienregulierung [37]. Entsprechend ist die Risikokommunikation parlamentarischer TA durch einen diskursöffnenden, informierenden und reflektierenden Ansatz gekennzeichnet, der nicht mit der Notwendigkeit konfrontiert ist, praxiswirksame Risikoentscheidungen kommunizieren zu müssen.

## Lehren aus früheren Risikodebatten

Risikokommunikation, so eine der Erfahrungen der TA mit vor allem der sozialen Dimension des Risikos, hängt stark von der jeweiligen Risikokonstellation ab [15]. Von daher kommt einer sorgfältigen Analyse dieser Konstellation hohe Bedeutung für eine gelingende Risikokommunikation zu. Ein Blick auf vergangene Risikodebatten macht das Spektrum deutlich [15].

In der Risikodebatte zur Kernenergie standen auf der einen Seite Entscheider aus Politik und Wirtschaft, unterstützt durch Experten aus Natur- und Technikwissenschaften. In Frontstellung dazu befanden sich auf der anderen Seite Betroffene, vor allem Anwohner an Standorten für geplante oder dann realisierte Kernkraftwerke oder andere kerntechnische Anlagen wie der geplanten Wiederaufbereitungsanlage Wackersdorf und am lange Zeit verfolgten Endlager Gorleben. Ihr Protest weitete sich auf beträchtliche Teile der deutschen Bevölkerung aus.

Die frühe Kernenergiedebatte (nicht nur) in Deutschland war geprägt von einer Arroganz der Experten gegenüber Kritik und Sorgen aus der Bevölkerung [38]. Gesellschaftliches Vertrauen in das Geflecht aus Experten, Wirtschaft und Politik, das hinter der Kernenergie stand, ging dadurch verloren, begleitet allerdings von einem generell größeren Misstrauen in den Staat. Auch das Vertrauen in das grundsätzliche Funktionieren demokratischer Prozesse und in das Expertentum in diesem Feld hat Schaden genommen, der teils bis heute andauert.

In der Debatte zur grünen Gentechnik verhielten sich die Dinge teils anders. Trotz der rhetorischen Fokussierung auf Risiken, z. B. die unkontrollierte Freisetzung gentechnisch veränderter Organismen, und trotz Fehler der Risikokommunikation, die teils durch das anhand der Kernenergie geschilderte Muster geprägt war, war das entscheidende Thema eher die soziale Dimension des Risikos, also die Verteilung von Risiken und Nutzen [26]. Der Endverbraucher, z. B. als Konsument von gentechnisch veränderten Nahrungsmitteln, hätte (anders als bei der Gentechnik im medizinischen Bereich) keinen ersichtlichen Nutzen von gentechnisch veränderten Nahrungsmitteln (außer vielleicht durch günstigere Preise, was aber in der Debatte keine Rolle spielte). Falls es allerdings zu gesundheitlichen Risiken käme (z. B. in Bezug auf Allergien), würden sich diese gerade beim Endnutzer zeigen. Aus deren Sicht ist es eine schlechte Bilanz, vom Nutzen ausgeschlossen zu sein, aber möglichen Gefährdungen ausgesetzt zu werden.

Während die Nanotechnologie Ende des 20. Jahrhunderts meist von fast paradiesischen Erwartungen gekennzeichnet war, drehte sich die Lage mit dem Aufkommen der Risikodebatte vor etwa 20 Jahren. Diese führte seitens Wissenschaft, Wirtschaft und Politik zu Befürchtungen, der Nanotechnologie könne es in Bezug auf die öffentliche Wahrnehmung ähnlich ergehen wie der Kernenergie oder der grünen Gentechnik [27, 34].

In der Tat kam es beispielsweise in Frankreich zu Demonstrationen gegen Nanotechnologie. Forderungen von zivilgesellschaftlichen Organisationen nach einer strikten Auslegung des Vorsorgeprinzips und nach einem Moratorium in Bezug auf Nanopartikel für marktgängige Produkte wie Kosmetika oder Lebensmittel (vgl. ETC-Group [36]) verschärften die Konfrontation. Dennoch ist die befürchtete fundamentalistische Verhärtung der Fronten nicht eingetreten. Ein Grund dürfte sein, dass die Risikokommunikation in diesem Fall sehr früh begonnen hat und von weitgehender Offenheit gekennzeichnet war. Sicher hat die offenere Verteilung von Chancen und Risiken im Vergleich mit der Gentechnik eine Rolle gespielt. Insbesondere jedoch haben Wissenschaftler und Wirtschaftsvertreter die Wissensdefizite über Risiken in diesem Feld von Beginn an nicht bestritten, sondern auf die Notwendigkeit von z. B. toxikologischer Forschung hingewiesen. Man könnte paradox formulieren: Weil auf allen Seiten offen über Nichtwissen und Risiken diskutiert wurde, wurden fundamentalistische Zuspitzung und Lagerbildung vermieden. Stattdessen blieb die Debatte konstruktiv. Das zunächst weitgehende Nichtwissen über Risiken von Nanopartikeln wurde in eine Forschungsaufgabe transformiert, mit Regulierungen auf vielen Ebenen, statt Verbote zu fordern und unmittelbare Opposition zu befördern.

Im Risikomanagement in Bezug auf öffentliche Kommunikation und die Informationspolitik sind diese Lehren einschlägig: Sie machen auf die Notwendigkeit von Vertrauen aufmerksam. Dies gilt auch für die Telemedizin, eines der aktuellen Themen des TAB [39]. Die Einbeziehung von Bürgern und Stakeholdern ist entscheidend für eine gesellschaftlich akzeptierte Risikokartierung, die gerade auch die soziale Dimension des Risikos ernstnimmt. Moderne Technikentwicklung und -verbreitung dürfen nicht in einer mehr oder weniger geschlossenen Welt der Ingenieure, Naturwissenschaftler und Manager betrieben werden, welche sich hinterher durch strategisch geschickte Kommunikation um die Akzeptanz ihrer eigenen Entwicklungen kümmern muss. Stattdessen sollten Entwicklung (jedenfalls in den Teilen, die keinen direkten Wettbewerbsinteressen der Firmen unterliegen) und Kommunikation in Transparenz und Offenheit erfolgen.

Stakeholder, zivilgesellschaftliche Organisationen und Bürger bzw. Patienten und Betroffene dürfen nicht erst zur Markteinführung, sondern müssen bereits in der Entwicklung und in vorbereitenden Schritten einer Technikeinführung einbezogen werden. Dies gibt zwar keine Garantie für einen konstruktiv-sachlichen Verlauf von Risikodebatten, dürfte jedoch in einer offenen und pluralen Gesellschaft eine notwendige Bedingung dafür sein.
